# MicroRNA miR-425 is a negative regulator of atrial natriuretic peptide

**DOI:** 10.1186/2050-6511-14-S1-O10

**Published:** 2013-08-29

**Authors:** Pankaj Arora, Connie Wu, Donald B Bloch, Brandi N Davis-Dusenbury, Ester Spagnolli, Akiko Hata, Sara Vandenwijngaert, Melissa Swinnen, Stefan Janssens, Emmanuel S Buys, Kenneth D Bloch, Christopher Newton-Cheh, Thomas J Wang

**Affiliations:** 1 Cardiology Division, Massachusetts General Hospital, Boston, Massachusetts, USA; 2 Cardiovascular Research Center, Massachusetts General Hospital, Boston, Massachusetts, USA; 3 Center for Human Genetic Research, Massachusetts General Hospital, Boston, Massachusetts, USA; 4 Program in Medical and Population Genetics, Broad Institute, Cambridge, Massachusetts, USA; 5 Department of Anesthesia, Critical Care, and Pain Medicine, Massachusetts General Hospital, Boston, Massachusetts, USA; 6 Division of Rheumatology, Allergy, and Clinical Immunology, Massachusetts General Hospital, Boston, Massachusetts, USA; 7 Center for Immunology and Inflammatory Diseases, Massachusetts General Hospital, Boston, Massachusetts, USA; 8 Stem Cell and Regenerative Biology, Harvard University, Cambridge, Massachusetts, USA; 9 Cardiovascular Research Institute, University of California, San Francisco, CA, USA; 10 Department of Cardiovascular Sciences, Gasthuisberg University Hospital, University of Leuven, Belgium

## Background

Numerous common genetic variants have been linked to blood pressure, but no underlying mechanism has been elucidated. Population studies have revealed that a genetic variant, rs5068 (A/G), is associated with blood pressure and the risk of hypertension. rs5068 lies in the 3’ untranslated region (3’UTR) of *NPPA*, the gene encoding atrial natriuretic peptide (ANP), and presence of the minor G allele is associated with increased circulating ANP levels and reduced blood pressure.

## Results

We hypothesized the existence of a microRNA (miR) that targets the *NPPA* 3’UTR and that the binding of the miR to the NPPA 3’UTR would be disrupted in transcripts from the rs5068 minor allele. We identified a microRNA, miR-425, that is predicted to bind the sequence spanning rs5068 for the A, but not the G, allele. miR-425 is expressed in human atria and ventricles. Using luciferase-3’UTR reporter constructs, we observed that miR-425 could silence reporter mRNAs carrying the *NPPA* major allele 3’UTR, but not those carrying the minor allele 3’UTR. Similarly, an anti-miR directed against miR-425 augmented expression of the luciferase-NPPA 3’UTR construct containing the major allele but not the minor allele. miR-425 reduced NPPA mRNA levels and ANP synthesis in human cardiomyocytes derived from induced pluripotent stem cells.

## Conclusion

Our studies provide mechanistic insights into how a common genetic variant identified in population genetic studies can regulate ANP levels and blood pressure. miR-425 is a novel regulator of ANP production, raising the possibility that miR-425 antagonists could be used to treat disorders of salt overload, including hypertension and heart failure.

